# Computer-Aided Detection for Breast Cancer Screening in Clinical Settings: Scoping Review

**DOI:** 10.2196/12660

**Published:** 2019-07-18

**Authors:** Rafia Masud, Mona Al-Rei, Cynthia Lokker

**Affiliations:** 1 Health Information Research Unit Department of Health Research Methods, Evidence, and Impact McMaster University Hamilton, ON Canada

**Keywords:** computer-aided detection, machine learning, screening mammography, breast cancer, radiology, implementation

## Abstract

**Background:**

With the growth of machine learning applications, the practice of medicine is evolving. Computer-aided detection (CAD) is a software technology that has become widespread in radiology practices, particularly in breast cancer screening for improving detection rates at earlier stages. Many studies have investigated the diagnostic accuracy of CAD, but its implementation in clinical settings has been largely overlooked.

**Objective:**

The aim of this scoping review was to summarize recent literature on the adoption and implementation of CAD during breast cancer screening by radiologists and to describe barriers and facilitators for CAD use.

**Methods:**

The MEDLINE database was searched for English, peer-reviewed articles that described CAD implementation, including barriers or facilitators, in breast cancer screening and were published between January 2010 and March 2018. Articles describing the diagnostic accuracy of CAD for breast cancer detection were excluded. The search returned 526 citations, which were reviewed in duplicate through abstract and full-text screening. Reference lists and cited references in the included studies were reviewed.

**Results:**

A total of nine articles met the inclusion criteria. The included articles showed that there is a tradeoff between the facilitators and barriers for CAD use. Facilitators for CAD use were improved breast cancer detection rates, increased profitability of breast imaging, and time saved by replacing double reading. Identified barriers were less favorable perceptions of CAD compared to double reading by radiologists, an increase in recall rates of patients for further testing, increased costs, and unclear effect on patient outcomes.

**Conclusions:**

There is a gap in the literature between CAD’s well-established diagnostic accuracy and its implementation and use by radiologists. Generally, the perceptions of radiologists have not been considered and details of implementation approaches for adoption of CAD have not been reported. The cost-effectiveness of CAD has not been well established for breast cancer screening in various populations. Further research is needed on how to best facilitate CAD in radiology practices in order to optimize patient outcomes, and the views of radiologists need to be better considered when advancing CAD use.

## Introduction

### Breast Cancer Screening

As the most commonly diagnosed cancer in women worldwide, breast cancer is a significant global health concern, representing about 25% of all cancer cases in 2012 [1]. It accounted for 522,000 deaths worldwide in 2012, ranking as the fifth leading cause of cancer-related death, and its incidence is higher in developing countries than in developed countries [1]. Breast cancer screening aims to detect cancer before the symptoms appear, with a goal of reducing mortality through early intervention [2]. Mammography is the most frequently used screening modality and can detect tumors before they become palpable and invasive [2].

Mammographic screening programs have been established in several developed countries. In 2015, the International Agency for Research on Cancer evaluated data from 40 combined studies in high-income countries in Europe, Australia, and North America and concluded that mammographic screening programs led to a 23% reduction in breast cancer mortality rates [3]. Although mammography has shown promising accuracy with only a single radiologist reading the images, 16%-31% of detectable cancers can be missed with this approach [4]. A second reading of the images by another radiologist, known as double reading, reduces the number of missed cases, resulting in an additional 3-11 cancers detected per 1000 women screened [4].

### Technology Adoption in Radiology

Technology is frequently adopted into health care practices to improve the quality of care delivered to patients. In radiology, technology adoption is common due to the field’s historical integration of clinical and technological facets. Broadly, artificial intelligence refers to the simulation of human intelligence, notably by computer systems, and includes the ability to learn and solve problems [5,6]. Machine learning is a subset of artificial intelligence and describes computer algorithms that “learn” how to perform tasks as they are exposed to data [7].

Radiology has immense potential to benefit from machine learning applications. McDonald et al [8] concluded that imaging volumes between 1999 to 2010 at one institution had disproportionately increased with the number of images that needed to be interpreted. Based on their study, an average radiologist in an 8-hour workday would need to interpret one image every 3-4 seconds to keep up with the surge in demand [8]. Human interpretation of clinical images has been shown to be a critical source of variability and error [9]. Factors such as incomplete pattern recognition and physical limitations such as fatigue can affect human interpretation of mammograms, while poor image quality and structure noise, which reduce visibility of low-contrast objects, can impede both human and computer interpretations [7].

### Computer-Aided Detection in Breast Cancer Screening

Advancements in computer algorithms are becoming increasingly sophisticated and widespread in the field of radiology, with the potential to be cost-effective for increasing detection rates of various medical conditions and improve the efficiency of radiologists [5]. One of the ways machine learning has been applied in breast imaging is through the use computer-aided detection (CAD) [10]. CAD can aid in the interpretation of medical images by serving as a double check or “second pair of eyes,” replacing the traditional double reading by a second radiologist [10,11]. CAD scans digital mammograms and marks suspicious areas of potential cancer features including masses and microcalcifications [10]. Radiologists generally review these marks after making their own interpretations and compare the two to reach a final assessment of the image [10]. The intended outcome is to reduce detection errors by the radiologist and increase the detection of cancers in the early stage, as this has a significant impact on breast cancer survival rates [11].

Although CAD has been approved for clinical use in mammography interpretation since 1998, its implementation in clinical settings has only recently spread [12]. In the United States, the use of CAD with digital screening mammograms increased dramatically from 5% in 2003 to 83% in 2012 [13]. With the prevalence of CAD, however, the perceptions of radiologists, who are the end users of CAD, have been largely overlooked in the debate of the diagnostic accuracy of CAD.

### Diagnostic Characteristics of Computer-Aided Detection

The goal of CAD is to increase the accuracy of breast cancer detection rates by increasing sensitivity, which will support radiologists in their diagnosis decisions [10]. CAD has the potential for use with a single reader, to match the performance of two readers in double reading, which saves radiologists’ time [14] and can be cost-effective [15]. As such, CAD with a single reader can be an alternative to double reading [16]. Although intended to increase cancer detection rates, many studies have published conflicting results, with some studies supporting the increased detection rates, while others showing no difference in detection rates and increased costs as compared to double reading [14,17]. The general consensus is that CAD provides some improvement in breast cancer detection, with up to 20% improvement in detection rates [16]. A recently published systematic review on the accuracy of CAD in screening mammography reported increased sensitivity in most studies adding CAD to single readings and no difference in sensitivity between double reading and single reading with CAD, with associated increases in recall rates when CAD was added to single reading [17].

### Implementation Factors

Implementation science is a scientific discipline that studies the methods to effectively integrate research findings into clinical practices [18]. Often, interventions in research are shown to be effective but they are not integrated into clinical settings to produce meaningful patient care outcomes [18]. There are various levels of health care delivery where barriers to implementation can occur, including the patient level, the provider level, and the policy level [19]. Other factors that can affect implementation include evidence quality, adaptability, and cost [18]. Self-efficacy is also important to consider for implementation, as individual beliefs and confidence can affect how one embraces change [18].

### Objective

As we continue to head into an artificial intelligence era, it is essential that we understand the implementation of technologies such as CAD in health care settings and its impact on health care providers and their potentially shifting roles. The objective of this review is to summarize the literature on the adoption and implementation of CAD for breast cancer detection, identify the barriers and facilitators to implementation, highlight knowledge gaps, and propose future research.

## Methods

This review followed the scoping review methodology proposed by Arksey and O’Malley [20] and advanced by Levac et al [21]. A scoping review investigates the breadth of a research topic, summarizes findings, and identifies gaps in existing literature [20]. MEDLINE was searched using Medical Subject Heading terms and text words related to breast cancer, imaging modalities, and implementation of CAD (Multimedia Appendix 1). We only searched MEDLINE, as it sufficiently covers the field of radiology practice. Although literature in the computer science and engineering fields may be relevant, they are usually focused on the technical development and accuracy of the technology, not implementation. Searches were completed up to March 2018. We limited our search to begin from 2010 in order to focus on recent advancements in CAD implementation, as deep learning has become more feasible and integrated into software services and applications. Only peer-reviewed papers in English were considered. Initial abstract screening was performed in duplicate by two independent reviewers. Full-text screening was performed in duplicate, with a third person acting as an adjudicator. Inclusion criteria were CAD for breast cancer screening applied to any imaging modality (eg, magnetic resonance imaging, digital mammography, and ultrasound) and use of at least one machine learning classifier. Original articles needed to focus on implementation, adoption, barriers, or facilitators for CAD use in a clinical setting. Articles that focused on accuracy of CAD or only described the machine learning algorithm or methodological approach were excluded. Reference lists and cited references in the included studies were also reviewed.

Data were charted based on the following characteristics: authors, year of publication, country of study, study methods, objective, and key results. Articles were tabulated in order of topic similarity including CAD use, CAD effect on reading time, and cost-effectiveness of CAD.

## Results

### Studies

**Figure 1:**
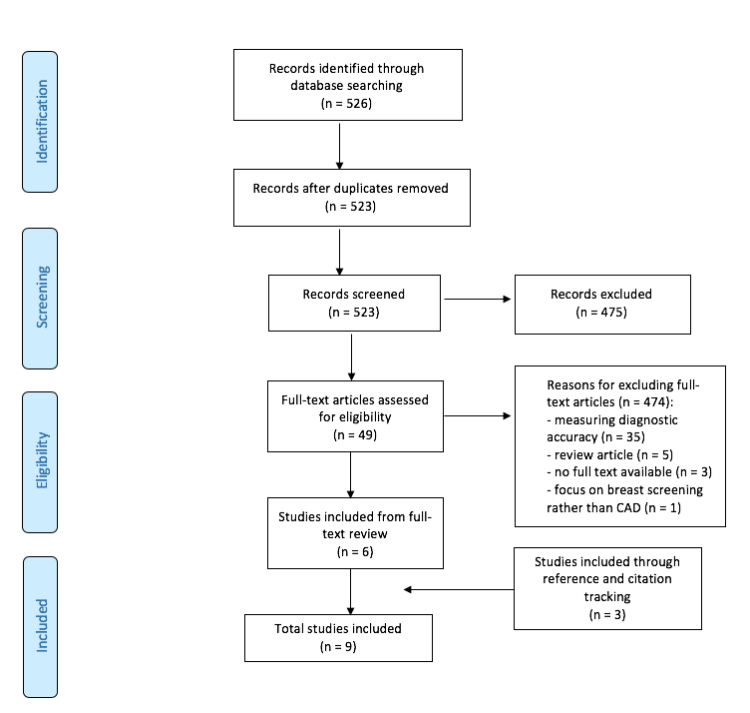
Preferred Reporting Items for Systematic Reviews and Meta-Analyses diagram of article selection. CAD: computer-aided detection.

Of the 526 articles identified by the initial search, 6 articles met the inclusion criteria and 3 other articles were included through reference and citation tracking [10,14,15,22-27] (Figure 1). Data extraction focused on the methods, objectives, and results of each included study (Table 1).

### Summary of Included Studies

The included articles used a range of methods (Table 1) including surveys of use and perceptions [10,25], retrospective analysis to determine the level of use and costs [22-24], prospective comparison of reading strategies [26,27], and cost-effectiveness analyses [14,15]. The objectives of the studies were widely variable, and only the study by Onega et al [25] addressed issues of CAD implementation for screening mammography directly by assessing radiologists’ perceptions of CAD. From the identified articles, themes that could affect implementation and uptake were generated and described. The themes were CAD prevalence, radiologist perceptions and confidence levels, interpretation times and recall rates, and the costs of CAD implementation.

### Computer-Aided Detection Prevalence

CAD use has increased over double reading since 2001 and remained stable in mammography practices in the United States between 2008 and 2016 [10,17,22,23]. Although the proportion of mammography screening volumes increased only slightly by about 2% from 2004 to 2008, the use of CAD screening increased by 91% in the same time period [24]. CAD was also used more by private offices (81%) compared to hospitals (70%) for screening mammography [24]. Incentives for CAD uptake include improved cancer detection rates [15,16,17,28], breast imaging profitability, and less radiologist time taken [25]. The use of CAD is also associated with a greater incidence of ductal carcinoma in situ and invasive breast cancer detected at earlier stages [22].

### Radiologist Perceptions and Confidence Levels

Onega et al [25] concluded that radiologists had overall more favorable perceptions of double reading by a colleague rather than single reading with CAD. Although this bias was present, three quarters of the 257 surveyed radiologists reported no use of double reading in their own practices [25]. Tchou et al [26] found that radiologists’ confidence levels in the use of CAD were mixed; however, confidence more often increased than decreased. The use of CAD led to changes in radiologists’ confidence in 22% (n=59) of the 267 cases, with confidence levels increasing in 14% (n=38) of the cases and decreasing in 8% (n=21) of cases; however, the use of CAD led to a change in radiologists’ conclusions in only 2% (n=5) of the cases [26].

### Interpretation Time and Recall Rates

Although CAD may take less time than double reading by a second radiologist, Tchou et al [26] found that reviewing CAD-marked images increased the mean interpretation time by 19%. The interpreting radiologist was also found to be a significant variable affecting the interpretation time of CAD-marked images [26]. Use of CAD concurrently with digital breast tomosynthesis increased the reading time by 29.2%, while reader interpretation performance was maintained [27]. Further, CAD implementation for breast cancer screening has been associated with a significant increase in recall rates, which is when a patient is called back for follow-up imaging [10,26]. Tchou et al [26] found an 11% increase in recall rates when CAD was used to interpret mammograms.

**Table 1:** Summary of recent studies on the implementation of computer-aided detection in clinical settings for breast cancer detection.

Author, year, country	Methods	Objectives	Results
Keen et al, 2018, United States [10]	Telephone surveys (400 digital mammography practices)	To assess whether CAD^a^ use by digital mammography practices decreased from 2008 to 2016	CAD use remained stable from 2008 to 2016 at US digital mammography practices (91.4% in 2008, 90.2% in 2011, and 92.3% in 2016).
Fenton et al, 2013, United States [22]	Retrospective cohort study of Medicare enrollees from the Surveillance, Epidemiology, and End Results Medicare database (409,459 mammograms and 163,099 women)	To study the relationship between CAD use and DCIS^b^ incidence and invasive breast cancer	CAD prevalence increased from 3.6% to 60.5% from 2001 to 2006, respectively. CAD use was linked to greater DCIS incidence. There was no difference in invasive breast cancer incidence; however, invasive breast cancer at earlier stages (I to II vs III to IV) was diagnosed.
Killelea et al, 2014, United States [23]	Retrospective cohort study of Medicare enrollees from the Surveillance, Epidemiology, and End Results Medicare database 2001-2002 (n=137,150) and 2008-2009 (n=133,097)	To evaluate the impact of CAD on screening-related cost and outcomes	CAD use increased from 3.2% to 33.1% from 2001-2002 to 2008-2009, respectively; however, a clinically significant change in stage at diagnosis was not observed.
Rao et al, 2010, United States [24]	Retrospective analysis of nationwide Medicare Part B fee-for-service databases from 2004 to 2008	To compare mammography procedure volumes and CAD use for (1) screening vs diagnostic mammography and (2) hospital facilities vs private offices	CAD was used for 74% of screening mammograms and 50% of diagnostic mammograms by 2008. CAD was used for 70% of hospital-based and 81% of private office-based screening mammograms.
Onega et al, 2010, United States [25]	Cross-sectional survey on the use and perceptions of CAD and double reading by radiologists (n=257)	To examine (1) the rates of CAD and double reading use for mammography interpretation and (2) the perceptions of CAD in comparison to double reading for mammography interpretation	More radiologists perceived that double reading improved cancer detection rates over CAD (74% vs 55% respectively). More than 75% use CAD for some screening mammography interpretation. 72% do not use double reading for screening mammograms.
Tchou et al, 2010, United States [26]	Prospective observational study of radiologists interpreting images with and without CAD (5 radiologists and 267 cases)	To study the effect of CAD on (1) interpretation time for reviewing CAD images, (2) recall rates, and (3) confidence levels	Use of CAD to interpret mammographic images resulted in a 19% or 23 second mean increase in interpretation time and 11% increase in recall rates. Confidence levels of radiologists were altered in 22% of cases: increased confidence in 14% and decreased confidence in 8%.
Benedikt et al, 2018, United States [27]	Prospective study multireader multicase crossover design of images (20 radiologists and 240 cases)	To compare reading time and performance with and without CAD, with concurrent use of DBT^c^	Concurrent use of CAD with DBT resulted in 29.2% faster reading time while maintaining reader interpretation performance.
Guerriero et al, 2011, United Kingdom [14]	Cost-effectiveness analysis (n=31,057)	To study the cost-effectiveness of single reading plus CAD versus double reading for women having routine screening across low-, average-, and high-volume units	Single reader with CAD is unlikely to be cost-effective, and savings from reading time would be offset by staff training Purchase, upgrading, and maintenance costs involved. Increased cost of assessment, although the model is sensitive to parameters that could change
Sato et al, 2012, Japan [15]	Cost-effective analysis using ICER^d^ ratio	To examine the cost-effectiveness of double reading by two readers versus single reading with CAD	Single reading with CAD for mammography screening is more cost-effective than double reading; results are sensitive to the number of examinees.

^a^CAD: computer-aided detection.

^b^DCIS: ductal carcinoma in situ.

^c^DBT: digital breast tomosynthesis.

^d^ICER: incremental cost-effectiveness ratio.

### Costs of Computer-Aided Detection Implementation

The implementation of CAD for breast cancer screening in clinical settings is associated with a significant financial cost. Rao et al [24] reported that Medicare spent US $33,706,444 on breast cancer screening fees for CAD in 2008. In the United Kingdom, replacing double reading with a single reader plus CAD cost an additional £227 per 1000 women in high-volume units, £253 per 1000 women in average-volume units, and £590 per 1000 women in low-volume screening units [14]. The overall cost of implementing CAD in the United Kingdom including assessment costs, equipment costs, and staff training was found to be greater than the savings in reading costs [14]. In Japan, the expected cost of implementing single reading with CAD is ¥2704 greater than that for double reading [15]. Cost-effectiveness analysis indicates that the use of CAD may be cost-effective, but it may vary depending on the accuracy of CAD, the number of patients screened, and comparison with single vs double reading [14,15].

## Discussion

### Principal Findings

Through our scoping review of the adoption and implementation of CAD in clinical settings for breast cancer detection and other related articles, CAD use by radiologists is based on trade-offs between the barriers and facilitators. The facilitators of CAD use for breast cancer screening include increased CAD uptake due to improved detection rates, increased profitability (in some contexts), and time saved from double reading [10,22-25]. The barriers include less favorable perceptions of CAD by radiologists, increased recall rates, increased costs, and an uncertain effect on patient outcomes [14,15,25-27].

### Facilitators for Computer-Aided Detection Use

Our results show that CAD use in mammography practices in the United States has increased dramatically in recent years and has remained stable to date [10,22-24]. Although not included in the scope of our review, since we excluded studies on the accuracy of breast cancer detection, several studies have shown an improvement in detection rates when shifting from traditional double reading or conventional mammography to CAD, with earlier detection of smaller tumors [11,16,28]. The use of CAD has specifically been linked to a significant increase in the detection rate of microcalcifications as well as an increase in the detection of ductal carcinoma in situ [13,22,28]. A 19.5% increase in the breast cancer detection rate is one of the highest reported increases with CAD implementation [29].

Based on a survey of radiologists [25], other reasons for the increase in CAD use over double reading includes greater profitability of breast imaging and less time taken by CAD. The rapid diffusion of CAD in the United States may be associated with the additional reimbursement for CAD, which is about US $7 per image by Medicare and more than US $20 per image from private insurers [10,13,25,30]. In addition to not being reimbursed, double reading takes up more time of radiologists compared to a single reader with CAD [25]. In settings such as Japan, where there is a shortage of radiologists for double reading and a need to increase breast cancer screening programs, the implementation of CAD as a second reader is appealing [15]. In Japan, Sato et al [15] found that single reading of mammograms with CAD was more cost-effective than double reading, especially when the screening volumes were high.

### Barriers for Computer-Aided Detection Use

Although CAD use has spread rapidly and double reading has declined in mammography practices in the United States, Onega et al [25] found that the surveyed radiologists had more favorable perceptions of double reading than CAD: 74% of the surveyed radiologists perceived double reading to improve cancer detection rates compared to 55% for CAD and 81% perceived that double reading reassures mammographers compared to 65% for CAD. Another barrier for CAD use is an increase in recall rates [26,27], which leads to unnecessary return visits. Tchou et al [26] found that of 33 recalls, only 4 (12%) resulted in a confirmed cancer diagnosis, while the rest were false-positives. Moreover, Keen et al [10] found through three national surveys that CAD decreases performance by increasing recall rates and decreasing the detection of invasive carcinoma while increasing the detection of ductal carcinoma in situ, whose detection value is debatable.

As with any technology, implementation of CAD is costly and may not always be cost-effective. In the United Kingdom, Guerriero et al [14] found that the costs associated with CAD, including equipment, training, and increased assessment costs outweighed the savings in reading costs, regardless of the screening volume. They concluded that compared to double reading, single reading with CAD was unlikely to be cost-effective without improvements in CAD effectiveness such as decreased recall rates [14].

Although several studies show increased detection rates, there is still some controversy regarding patient outcomes with the use of CAD for screening mammograms because some studies have reported conflicting results [13,31]. A study on detection rates [13] found no evidence of increased breast cancer detection rates with CAD as compared to those without CAD and concluded there is no established added benefit with CAD. Romero et al [28] found that detection rates increased with CAD, but the increase was not statistically significant. Killelea et al [23] found that the detection of early stage tumors with CAD was not significant. Bargolla et al [16] found that CAD did not detect any cancer that the radiologist did not initially perceive. Furthermore, the findings of Gross et al [32] in the United States suggest that the use of CAD or digital mammography has limited effectiveness for older, average-risk women and that higher costs associated with the adoption of such technologies may not necessarily lead to better outcomes.

### Trade-Offs for Computer-Aided Detection Use

The use of CAD for breast cancer screening involves several tradeoffs including weighing the impact on detection rates and patient outcomes, costs and financial incentives, time saved from double reading, increased recall rates, and radiologist perceptions. The majority of our included studies were based in the United States, where Medicare reimbursement for CAD images provides a financial incentive for uptake. Although the clinical impact of CAD on patient outcomes is not agreed upon, CAD use has increased and remained stable in the United States [10,22,23,24]. CAD reimbursement was a crucial part of marketing that manufacturers used to target mammography practices [22]. This partly explains why CAD use has prevailed in the United States, despite Onega et al [25] showing that most of the surveyed radiologists perceived double reading more favorably over CAD.

In other countries, the tradeoffs of using CAD for breast cancer screening can vary and cost-effectiveness must be assessed independently. In our included studies, we found that cost-effectiveness of CAD for breast cancer screening was formally assessed in the United Kingdom and Japan [14,15]. Although implementation of CAD was reported to be more cost effective than double reading in Japan [15], it was unlikely to be cost-effective in the United Kingdom [14]. Before investing in the widespread use of CAD in mammography practices in a specific context, its cost-effectiveness should be thoroughly evaluated while weighing the barriers against the facilitators.

### Implications of Computer-Aided Detection Use on Radiology Practices

The introduction of machine learning applications such as CAD for mammogram screening is changing modern radiology practice [33]. Some recent articles suggest that artificial intelligence and machine learning pose a major threat to radiologists [34,35]. In contrast, others such as Recht and Bryan [33] and Dreyer and Geis [5] stand by the view that advancements in artificial intelligence and machine learning will be a milestone for radiologists and will increase their efficiency by allowing them to carry out more “value-added tasks” such as more extensive patient interaction and integrated care. They argue that machines are not able to perform these “value-added tasks”; therefore, they are not a threat to replacing radiologists and will rather make them “better radiologists” [33]. Tang et al [6] distinguished between tasks and work of radiologists and described aspects of radiologists’ complex work that cannot be done by artificial applications, including integration of knowledge from scientific fields and clinical specialties for explaining certain images, quality control, disease monitoring, interventional procedures, etc [6]. Through our review on the implementation of CAD in breast cancer screening, we did not find any studies evaluating the redistribution of tasks among radiologists to support this suggestion. Future research could assess the effect of CAD on radiologists’ workflow and tasks.

### Limitations

This scoping review is limited by the low number of included publications. Most articles detected in our initial database search (Figure 1) were excluded, as they focused on the diagnostic accuracy of CAD rather than the implementation of CAD, although we recognize the value of high accuracy as a requirement for implementation and adoption. We searched only MEDLINE, which would have limited the detection of articles from the fields of computer science and engineering; this was a deliberate choice because MEDLINE covers the fields of radiology and implementation science. Three of the nine included articles were not detected in our searches but were found through reference checking. Our search strategy included truncated textwords for adoption and as implementation terms, which would have limited our retrieval, as these terms are used inconsistently in the implementation science field but were added to our searches to improve specificity.

### Conclusions

This review is important in summarizing the recent evidence of facilitators and barriers for CAD implementation in the literature and acknowledging any gaps. Our review suggests that there is a large focus on the diagnostic accuracy of CAD, but little focus on CAD implementation and perceptions of radiologists—the end users. With the increasing prevalence of CAD in mammography practices, especially in the United States, it is important to understand how CAD impacts radiologists, their practice, and the health care system. Although there is a financial incentive for radiologists to use CAD in the United States, it is still unclear whether better patient outcomes are being achieved. The tradeoffs of implementing CAD in different settings should be considered, especially the cost-effectiveness, as there is a significant investment involved in the transition to CAD. Lastly, it is important to continue to consider the perceptions of radiologists, who are the end users of CAD.

We propose that further studies be carried out to better understand CAD adoption and implementation in clinical settings. Specifically, there should be a focus on investigating radiologists’ perceptions of CAD use in various settings, as we only came across one such study based in the United States, which cannot be generalized to other settings and health care systems. In addition, a better understanding of the extent to which CAD is used in different countries and policies that have led to these levels of use can be explored. Lastly, the cost-effectiveness of CAD use for breast cancer screening in various populations should be assessed to determine appropriate thresholds in order to facilitate CAD implementation.

## Appendix

Multimedia Appendix 1 Search terms.

## Abbreviations

CAD: computer-aided detection
DBT: digital breast tomosynthesis
DCIS: ductal carcinoma in situ
ICER: incremental cost-effectiveness ratio
