# Correction: Computer-Aided Detection for Breast Cancer Screening in Clinical Settings: Scoping Review

**DOI:** 10.2196/15799

**Published:** 2019-08-21

**Authors:** Rafia Masud, Mona Al-Rei, Cynthia Lokker

**Affiliations:** 1 Health Information Research Unit Department of Health Research Methods, Evidence, and Impact McMaster University Hamilton, ON Canada

In “Computer-Aided Detection for Breast Cancer Screening in Clinical Settings: Scoping Review” (JMIR Med Inform 2019;7(3):e12660) in the Results section under the subheading “Interpretation Time and Recall Rates”, the phrase “Use of CAD concurrently with digital breast tomosynthesis increased the reading time by 29.2%....” has been replaced by “Use of CAD concurrently with digital breast tomosynthesis reduced the reading time by 29.2%...”.

The correction will appear in the online version of the paper on the JMIR website on August 21, 2019, together with the publication of this correction notice. Because this was made after submission to PubMed, PubMed Central, and other full-text repositories, the corrected article also has been resubmitted to those repositories.

