# Supplementary material for: Computer-Aided Detection for Breast Cancer Screening in Clinical Settings: Scoping Review
Source: JMIR Med Inform. 2019 Jul 18;7(3):e12660. doi: 10.2196/12660 (PMC6670274; doi:10.2196/12660)
Supplement: Multimedia Appendix 1 [file medinform_v7i3e12660_app1.pdf]

Appendix

MEDLINE search terms

1. mammography/
2. mammography.tw.
3. diagnostic imaging/
4. diagnostic imaging.tw.
5. mass screening/
6. mass screening.tw.
7. Ultrasonography, mammary/
8. Ultrasonography, mammary.tw.
9. Decision Support Systems, Management/
10. Decision Support Systems, Management.tw.
11. Radiology Information Systems/
12. Radiology Information Systems.tw.
13. Technology, Radiologic/
14. Technology, Radiologic.tw.
15. 1 or 2 or 3 or 4 or 5 or 6 or 7 or 8 or 9 or 10 or 11 or 12 or 13 or 14
16. machine learning/
17. machine learning.tw.
18. exp algorithm/
19. algorithm.tw.
20. Automatic Data Processing/
21. Automatic Data Processing.tw.
22. computer aided detection.tw.
23. artificial intelligence.tw.
24. 16 or 17 or 18 or 19 or 20 or 21 or 22 or 23
25. 15 and 24
26. exp breast neoplasms/
27. breast neoplasm*.tw.
28. breast cancer.tw.
29. 26 or 27 or 28
30. 25 and 29
31. adopt*.tw.
32. implement*.tw.
33. barrier*.tw.
34. accept*.tw.
35. improve*.tw.
36. benefit*.tw.
37. challenge*.tw.
38. performance*.tw.
39. utiliz*.tw.
40. facilitat*.tw.
41. 31 or 32 or 33 or 34 or 35 or 36 or 37 or 38 or 39 or 40

42. 30 and 41
